# Supplementary material for: Decrease in Socioeconomic Disparities in Self-Rated Oral Health among Brazilian Adults between 2013 and 2019: Results from the National Health Survey
Source: Int J Environ Res Public Health. 2024 Sep 10;21(9):1198. doi: 10.3390/ijerph21091198 (PMC11431346; doi:10.3390/ijerph21091198)
Supplement: Supplementary file 1 [file ijerph-21-01198-s001.zip › ijerph-3156409-supplementary.pdf]

International Journal of
Environmental Research and Public Health

MDPI

Article

Decrease of socioeconomic disparities in self-rated oral health among Brazilian adults between 2013 and 2019: results from the National Health Survey

Anna Rachel dos Santos Soares [1], Maria Luiza Viana Fonseca [1], Deborah Carvalho Malta [2], Loliza Luiz Figueiredo Houri Chalub[1] and Raquel Conceição Ferreira [1]*

Supplementary Materials: The following supporting information can be downloaded at www.mdpi.com/xxx/s1, Table S1: Module and questions of the NHS and the variables analyzed; Figure S1: Odds Ratio and 95% Confidence Interval of factors associated with SROH (NHS 2013); Figure S2: Odds Ratio and 95% Confidence Interval of the factors related with SROH (NHS 2019).

Table S1. Module and questions of the NHS and the variables analyzed.

MODULE	QUESTION	VARIABLE IN THIS STUDY
C – General Characteristics of Residents	C006	Sex
	C008	Age
D - Characteristics of Education of people aged 5 or over	D001, D002, D003, D008, D009	Education
E - Job of household residents	E01602, E01604, E01802, E01804	Income
F - Household Income	F00102, F00702, F00802	
U – Oral Health	U005	Self – Rated Oral Health (SROH)

Table S2. Odds Ratio and 95% Confidence Interval of factors associated with SROH (NHS 2013) (MW: minimum-wage; Yrs: Years of study; Yrs old: Years old).

Variables	OR	95% CI
Sex		
Male	1	
Female	1.18***	1.10 – 1.25
Age group (years old)		
18 – 24	1	
25 – 39	0.83***	0.74 – 0.92
40 – 59	0.60***	0.53 – 0.66
≥ 60	0.60***	0.53 – 0.68
Education (years of study)		
0 – 4	1	
5 – 8	1.02	0.94 – 1.12
9 – 11	1.32***	1.20 – 1.46
≥ 12	2.01***	1.77 – 2.28
Income (minimum wage)		
0 – 1	1	
1.1 – 2	1.41***	1.30 – 1.52
2.1 – 3	1.60***	1.41 – 1.80
≥ 3.1	2.47***	2.16 – 2.83

Brazilian Regions		
North	1	
Northeast	1.00	0.92 – 1.01
Southeast	1.55***	1.41 – 1.71
South	1.55***	1.38 – 1.74
Midwest	1.30***	1.18 – 1.44

Note: Exponentiated coefficients; * $p < 0.05$, ** $p < 0.01$, *** $p < 0.001$.

Table S3. Odds Ratio and 95% Confidence Interval of factors associated with SROH (NHS 2019) (MW: minimum-wage; Yrs: Years of study; Yrs old: Years old).

Variables	OR	95% CI
Sex		
Male	1	
Female	1.61***	1.10 – 1.22
Age group (years old)		
18 – 24	1	
25 – 39	0.82***	0.74 – 0.91
40 – 59	0.63***	0.57 – 0.70
≥ 60	0.62***	0.55 – 0.70
Education (years of study)		
0 – 4	1	
5 – 8	0.86**	0.79 – 0.95
9 – 11	1.15**	1.05 – 1.27
≥ 12	1.52***	1.32 – 1.76
Income (minimum wage)		
0 – 1	1	
1.1 – 2	1.38***	1.20 – 1.59
2.1 – 3	1.37*	1.12 – 1.69
≥ 3.1	1.88***	1.41 – 2.50
Brazilian Regions		
North	1	
Northeast	0.83***	0.77 – 0.89
Southeast	1.11*	1.03 – 1.21
South	1.20***	1.09 – 1.31
Midwest	0.97	0.89 – 1.06
Interaction (income # education)		
1.1 – 2 MW # 0 – 4 yrs of study	1	
1.1 – 2 MW # 5 – 8 yrs of study	0.99	0.82 – 1.21
1.1 – 2 MW # 9 – 11 yrs of study	1.12	0.96 – 1.30
1.1 – 2 MW # ≥ 12 yrs of study	1.15	0.90 – 1.46
2.1 – 3 MW # 0 – 4 yrs of study	1	
2.1 – 3 MW # 5 – 8 yrs of study	1.68**	1.20 – 2.33
2.1 – 3 MW # 9 – 11 yrs of study	1.32*	1.03 – 1.74
2.1 – 3 MW # ≥ 12 yrs of study	1.29	0.98 – 1.71
≥ 3 MW # 0 – 4 yrs of study	1	
≥ 3 MW # 5 – 8 yrs of study	1.10	0.73 – 1.68
≥ 3 MW # 9 – 11 yrs of study	0.96	0.67 – 1.38
≥ 3 MW # ≥ 12 yrs of study	1.45*	1.04 – 2.02

Note: Exponentiated coefficients; * $p < 0.05$, ** $p < 0.01$, *** $p < 0.001$.
